# Sound–action symbolism in relation to precision manipulation and whole-hand grasp usage

**DOI:** 10.1177/17470218231160910

**Published:** 2023-03-21

**Authors:** Lari Vainio, Markku Kilpeläinen, Alexandra Wikström, Martti Vainio

**Affiliations:** 1 Phonetics and Speech Synthesis Research Group, Department of Digital Humanities, University of Helsinki, Helsinki, Finland; 2 Perception, Action & Cognition Research Group, Department of Psychology and Logopedics, Faculty of Medicine, University of Helsinki, Helsinki, Finland

**Keywords:** Sound symbolism, grasping, speech, utilisation, pantomimes

## Abstract

It has been suggested that actions can provide a fruitful conceptual context for sound symbolism phenomena, and that tight interaction between manual and articulatory processes might cause that hand actions, in particular, are sound-symbolically associated with specific speech sounds. Experiment 1 investigated whether novel words, built from speech sounds that have been previously linked to precision or power grasp responses, are implicitly associated with perceived actions that present precision manipulation or whole-hand grasp tool-use or the corresponding utilisation pantomimes. In the two-alternative forced-choice task, the participants were more likely to match novel words to tool-use actions and corresponding pantomimes that were sound-symbolically congruent with the words. Experiment 2 showed that the same or even larger sound–action symbolism effect can be observed when the pantomimes present unfamiliar utilisation actions. Based on this we propose that the sound–action symbolism might originate from the same sensorimotor mechanisms that process the meaning of iconic gestural signs. The study presents a novel sound–action phenomenon and supports the view that hand–mouth interaction might manifest itself by associating specific speech sounds with grasp-related utilisations.

## Introduction

Although the relationship between the sound of a word and its meaning is predominately arbitrary in the lexicon (e.g., de Hockett, 1963; Saussure, 1916), many exceptions in the form of a nonarbitrary relationship have been recognised (Blasi et al., 2016). Onomatopoeic words, such as ring and knock in English, are the most obvious examples of this type of sound symbolic items of vocabulary. Linguistics and cognitive science have also documented many non-onomatopoeic associations between speech sounds and meanings in which the speech sound iconically represents some aspect of a referent object, such as colours (Johansson et al., 2020), emotions (Adelman et al., 2018), motion speed (Cuskley, 2013), and brightness (Hirata et al., 2011). The nonarbitrary linkages between speech sounds and meanings that have received the most attention from researchers associate specific speech sound with the size of the referent object (Sapir, 1929; Winter & Perlman, 2021) and shape (Knoeferle et al., 2017; Köhler, 1929; Shinohara et al., 2020). In general, the nonarbitrary associations between speech sounds and meaning have been proposed to play a role in the acquisition and evolution of language (Imai & Kita, 2014; Nielsen & Dingemanse, 2021).

In one type of sound symbolism, which has been relatively little researched, speech sounds are associated with actions. For instance, in Japanese, mothers use sound-symbolic mimetics, which is the word class of non-onomatopoeic ideophones, five times more often with a child than with an adult when referring to actions (Nagumo et al., 2006). In line with this observation, Imai et al. (2008) demonstrated that children as young as 2 years old are able to detect sound–action matches between novel sound-symbolic verbs and actions. The children were asked to watch two action videos on different types of walking and then judge which one of the two videos provided a better match to a novel verb that was constructed to sound-symbolically match only one of the actions.

The sound-symbolic phenomenon, which might be closely related to sound–action symbolism, is sound–movement symbolism. Although this phenomenon has not been investigated in many studies, in general, it can be stated that back vowels are associated with slower movements than front vowels (Cuskley, 2013). Regarding the movement shape, movements with acceleration and deceleration tend to be associated with obstruent sounds, while movements without much acceleration are associated with sonorant sounds (Shinohara et al., 2020; Shinohara et al., 2016). These investigations suggest that people might show a tendency to associate particular speech sounds with particular types of velocity and jerkiness profiles of a viewed movement. It is noteworthy that these movement features often vary in observed actions and can consequently contribute to sound–action symbolism phenomena.

The present study investigates whether a similar sound–action symbolism effect to that observed in relation to different manners of walking (Imai et al., 2008) can be also observed in relation to grasp-related utilisation. The rationale for the hypothesis that a similar sound–action symbolism effect would be observed in relation to manipulative hand movements (Vainio & Vainio, 2021) is based on theoretical views and empirical evidence suggesting that manual actions and speech processes might be integrated, at least to some extent. Regarding manual actions, grasping in particular has been linked to developmental and evolutionary aspects of language (Greenfield, 1991; Rizzolatti & Arbib, 1998). Concerning language evolution, Arbib (2005) provided a framework proposing that the evolution of an imitation system for grasping played a critical role in the emergence of vocal communication. This view has theoretical overlaps with what is called the gestural theories of speech evolution (Corballis, 2003; Kendon, 2017). Many versions of this theory highlight that vocal communication might have partially evolved from an involuntary tendency to use orofacial gestures to mimic manual gesticulations and manipulation actions (Hewes, 1973; Paget, 1944). The theories that assume a role of hand–mouth coupling in language evolution are supported by empirical evidence that demonstrates neural and functional interaction between mouth movements and grasping (Gentilucci et al., 2001; Rizzolatti et al., 1988).

In line with views that assume a tight neural and functional coupling between grasping and mouth actions, Ramachandran and Hubbard (2001) proposed that many instances of sound symbolism may involve cross-domain mappings between the motor processes related to articulatory and hand gestures, and an implicit tendency to conceptually associate the visual appearance of an object, such as size or shape, with the abstracted representations of these articulatory and hand gestures. For example, this position asserts that forming the front-close vowel when articulating words, such as *little* and *tiny*, mimics precision grip gestures that in turn are associated with small objects and concepts. Hence, this hypothesis can be understood as assuming that, for example, size–sound symbolism, which links front-close vowels with small magnitudes (Sapir, 1929; Winter & Perlman, 2021), might to some extent be based on automatically representing conceptual and perceptual magnitude information of a referent object in integrated articulatory and hand-related motor processes.

The account of Ramachandran and Hubbard is supported by evidence demonstrating that (a) representing perceived precision grip and objects that are graspable using the precision grip, automatically involves precision grip-related motor processes (Franca et al., 2012; Vainio et al., 2007) as well as processes associated with articulating the vowel [i] (Vainio et al., 2017, 2019) and that (b) response processes related to the precision grip and articulating front-close vowels are tightly integrated (Vainio et al., 2013). This latter evidence (b) was observed using a dual-action task in which participants were visually presented with an individual vowel or a consonant in a green or blue colour while they were holding a precision and power grip response device in their hand. Their task was to perform either precision (i.e., pinching the device between the tips of the index finger and thumb) or a power grip (i.e., squeezing the device using partly flexed fingers, thumb and the palm) response according to the colour, and simultaneously pronounce the presented vowel/consonant. This task has revealed that the high-front vowels ([i] and [y]) and the alveolar consonants ([d], [r], [s], and [t]) are associated with precision grip responses, while low and/or back vowels ([ɑ], [o], [u], and [æ]), as well as velar consonants or those consonants whose articulation involves the raising or lowering of the tongue body ([k], [l], and [m]), are associated with power grip responses (Vainio et al., 2018; Vainio & Vainio, 2022).

Most of the manipulation actions performed by a human hand can be divided into two categories: precision manipulation (PM) and whole-hand grasp usage (WHGU) (Bullock, 2016). The PM actions (e.g., writing and key insertion) involve PM in the fingertips—most often using the fingertips of the thumb and index finger—while the WHGU actions typically involve grasping, holding, and manipulating an object using the power grasp (e.g., slicing a bread and opening a jar). The above-mentioned theories of language evolution as well as empirical evidence concerning integration in processing articulatory gestures and grasp actions suggest that people may display a tendency to sound-symbolically associate novel verbs with PM actions if these verbs consist of speech sounds that have been linked to precision grip responses. Similarly, novel verbs consisting of speech sounds that have been linked to power grip responses could be associated with observed actions that present WHGU. To investigate this hypothesis, we administered the two-alternative forced-choice (2-AFC) task, which has been used widely in sound symbolism investigations (e.g., Margiotoudi et al., 2019; Margiotoudi & Pulvermüller, 2020). Most typically, in this task, participants are presented with two visual stimuli (e.g., a round vs sharp shape) and a visual or auditory word—novel or drawn from unfamiliar language—which is sound-symbolically congruent with one stimulus and incongruent with the other stimulus. Participants are then required to select which of the two stimuli provides a better match to the word. The sound symbolism manifests itself in that participants select the stimulus, which sounds symbolically matches the word above the chance level.

So far, there is very limited evidence for supporting the view proposed by Vainio and Vainio (2021) that certain observed actions can be sound-symbolically associated with particular speech sounds. As far as we know, the study by Imai et al. (2008) is the only sound-symbolic investigation that presents this interaction. In particular, there is no evidence that observed manipulative hand actions would be sound-symbolically associated with speech sounds. The present study aims to overcome these shortcomings. In this study, the 2-AFC task is used to explore whether novel words are associated with perceived PM and WHGU when the words are built from speech sounds that are hypothetically congruent with the precision or power grip. Accordingly, participants are presented with a novel word together with two video clips in which one presents PM (e.g., lighting a match) and the other presents WHGU (e.g., opening a jar). It is hypothesised that if the participants have a tendency to associate certain speech sounds with the actions of PM or WHGU, they should select the utilisation action that sound-symbolically matches the phonetic components of the word above the chance level.

It has been shown that vocalisation processes can be systematically influenced by perceiving graspable tools (Vainio et al., 2019). Hence, given that the stimuli of utilisation actions, used in the present study, contain hand movements as well as a graspable tool that is manipulated, for control purposes, in Experiment 1, in half of the stimulus conditions, the object is removed, and the manipulative hand action is performed as a utilisation pantomime. This experimental manipulation also presents a secondary research question: Are observed utilisation pantomimes sound-symbolically associated with speech sounds? Regarding pantomimes, it has been suggested (Osiurak et al., 2012) that recognising tool-use actions and utilisation pantomimes are based on somewhat different processes. This position proposes that, in general, recognition of utilisation actions is achieved to some extent by processing the kinematic movement parameters and hand shape as well as recognising the context of the movement. These context-related recognition processes might require, for instance, recognising an object that is used for the action. However, when recognising the utilisation pantomimes, people have to infer this contextual information solely from the kinematic movement parameters and hand shape. That is, regarding tool-use actions, the action could be recognised even from a still image of the action by processing the manipulated object and the hand posture that is used for the manipulation. In contrast, concerning the utilisation pantomimes, perceptual and contextual processing have to be dominantly directed to the kinematic movement parameters and hand shape, so that contextual information could be inferred from them. According to Osiurak et al., this leads to a weakened recognition of a pantomimic action in comparison to the tool-use action. Thus, for theoretical reasons, it is interesting to explore whether the hypothetical sound–action effect related to utilisation behaviour can be observed even when the object is missing from the stimulus and whether the effect is larger or smaller when the observed action is object-directed rather than pantomime. For example, if the effect would be larger in relation to pantomimes in comparison to corresponding tool-use actions, this would suggest that the sound–action symbolism effects might be based predominately on processing the kinematic movement parameters and/or the hand shape.

## Experiment 1

### Methods

#### Participants

Twenty-eight volunteers participated in Experiment 1 (21–42 years of age; mean age = 27 years; seven males; two left-handed). All participants had normal or corrected-to-normal vision and hearing. All participants were native Finnish speakers. In addition, some participants were able to communicate in the following languages: English, Swedish, French, Russian, Spanish, Italian, and Portuguese. None of them were familiar with any of the following languages: Japanese, Burmese, Indonesian, and Hawaiian. This aspect was important because the participants were misinformed that their task was to guess the meaning of words taken from these four languages (see below). As this study is our first attempt to explore the interaction between observed utilisation actions and speech sounds, we based our number of participants on the research by Imai et al. (2008; Experiment 1b) because it was methodologically and theoretically closest to our study. They similarly used the 2-AFC task to explore sound–action phenomenon. That study used 15 participants, and a somewhat smaller number of experimental trials than the present study, to show that the action, which is matching to the sound, is selected significantly (*p* < .01, *d* = 0.90) above the chance level. Based on this, the sample size calculation carried out using G*Power software (Faul et al., 2007), proposed that already 16 participants would suffice to produce a sound–action effect, which is significantly above chance, using a similar forced-choice matching task. Furthermore, while Imai et al. recruited 15 subjects for their study, we nearly doubled that sample size because our analysis contained one more factor (i.e., tool-use actions vs pantomimic action). After the experiment, the participants were asked whether they had previous knowledge of sound symbolic phenomena. One participant reported having previous knowledge about size–sound symbolism. The fact that 95.8% of her responses were sound-symbolically congruent indicated that her responses were most probably driven by her previous knowledge of size–sound symbolism. For this reason, her data were removed from the analysis. As a consequence, all of the 27 participants whose data were included in the analysis were naive to the purpose of the study; they appeared to be unaware of the purpose of the study and the nature of the investigated effect. We obtained written informed consent from all participants. The study was approved by the Ethical Review Board in Humanities and Social and Behavioural Sciences at the University of Helsinki.

#### The auditory and visual stimuli

*Auditory stimuli*: We created 24 novel words that were hypothesised to be sound-symbolically congruent with PM actions (”small” words), and 24 novel words that were hypothesised to be sound-symbolically congruent with WHGU actions (“large” words). Hence, the “small” and “large” words were constructed from the varying combination of vowels/consonants that have been previously associated, respectively, with the precision grip ([i], [y], [d], [r], [s], [t]) and power grip responses ([ɑ], [o], [u], [æ], [k], [l], [m]) (Vainio et al., 2013; Vainio et al., 2018; Vainio & Vainio, 2022). In addition to these vowels/consonants, given that [p] has been associated with small concepts, while [b], [v], and [g] have been associated with large concepts in the previous literature on sound symbolism (e.g., Berlin, 2006; Newman, 1933; Thompson & Estes, 2011), we included [p] in some ”small” words, while [b], [v], and/or [g] were included in some “large” words. The speech sound [ŋ] (i.e., <ng>) was also incorporated into some “large” words because it can be assumed to be linked to the power grip, as it is a velar speech sound (Vainio & Vainio, 2022). The letter <c> was included in the written forms of some “small” and “large” words such that in relation to “small” words, it was pronounced as [s] (i.e., as a precision grip-related consonant), while in relation to “large” words, it was pronounced as [k] (i.e., as a power grip-related consonant). The letter <z> was included in two “small” words because, in Finnish, it is pronounced as a combination of [t] and [s] (i.e., [ts]) that are both associated with the precision grip (Vainio & Vainio, 2022). Finally, the vowel [e] and consonant [h] were included as grip-neutral speech units in some “small” and “large” words because our previous research does not systematically associate them with the precision or power grip (Vainio et al., 2013; Vainio & Vainio, 2022).

Twelve words (six “small” and six “large”) loosely mimicked Japanese verbs, 12 (six “small” and six “large”) mimicked Indonesian verbs, 12 (six “small” and six “large”) mimicked Burmese verbs, and 12 (six “small” and six “large”) mimicked Hawaiian verbs. For example, we translated the word “*puristaa*” (squeeze) from Finnish into Japanese using Google Translate—a translation tool, and then we replaced some of the original letters, so that the letters were related either to the precision or power grip. These four languages were selected because they are typically unfamiliar to Finns (see the “Participants” section). Each “large” word was created for each language category, so that it needed to match the “small” words of the same language having the same number of letters in the same locations for the vowels and consonants (e.g., *izirsei* vs *agolmea*). As such, the “small” and “large” words included the same number of letters in general. Only the phonemes allowed by Finnish phonology and phonotactics were used. For the auditory stimuli, each word was intentionally articulated at the same tempo, intensity, and pitch by a male who was a native speaker of Finnish. The auditory stimuli were manipulated using Praat (6.2.10), so that the profile of the fundamental frequency, as well as intensity, was similar for every stimulus. The list of “small” and “large” words is presented in Table 1.

**Table 1.** The novel words organised by the size (%) of the congruency effect (the left percentage refers to congruent responses of Experiment 1 and the right percentage refers to congruent responses of Experiment 2).

Word	Language	Size	%	Word	Language	Size	%
hogemako	Japanese	Large	77.8/84.6	ieisiti hipite	Hawaiian	Small	63.0/69.2
amenaom’bulkam	Indonesian	Large	77.8/69.2	kuengkan memacu	Burmese	Large	63.0/69.2
halaleoko äleaka	Japanese	Large	77.8/84.6	okakea	Hawaiian	Large	63.0/88.5
iseniyt’pitsys	Indonesian	Small	77.8/80.8	situizi	Japanese	Small	63.0/80.8
itipe hisetsiteri	Japanese	Small	77.8/69.2	tesripi	Burmese	Small	59.3/61.5
lokema’gao	Burmese	Large	77.8/69.2	tentytsi	Indonesian	Small	59.3/61.5
wahame kabaka	Hawaiian	Large	74.1/88.5	tidsice	Indonesian	Small	55.6/69.2
ghaa ong’moahe	Burmese	Large	74.1/80.8	dhii yit’siyhe	Burmese	Small	55.6/61.5
hangagan koge	Indonesian	Large	74.1/80.8	ihi hie	Hawaiian	Small	55.6/53.9
keamomobe koago	Japanese	Large	74.1/69.2	ipie’tyty	Hawaiian	Small	55.6/80.8
Kengagu	Burmese	Large	74.1/57.7	izirsei	Japanese	Small	55.6/73.1
Loagakal	Burmese	Large	70.4/80.8	okue’läla	Hawaiian	Large	55.6/53.9
dihite tipite	Hawaiian	Small	70.4/53.9	diytytir	Burmese	Small	51.9/46.2
etihie itipisy	Hawaiian	Small	70.4/80.8	memalag lemomo	Indonesian	Large	51.9/80.8
hisridin rite	Indonesian	Small	70.4/73.1	mengangu	Indonesian	Large	51.9/92.3
Isytei	Hawaiian	Small	66.7/76.9	oha hae	Hawaiian	Large	51.9/84.6
mangakha	Indonesian	Large	66.7/92.3	akabe havelmokeko	Japanese	Large	48.2/65.4
Agolmea	Japanese	Large	66.7/80.8	oealaka hobake	Hawaiian	Large	48.2/76.9
diteti’syi	Burmese	Small	66.7/80.8	shirteri’tis tei	Burmese	Small	48.2/57.7
evahae akabuga	Hawaiian	Large	66.7/92.3	sitsirhi	Indonesian	Small	48.2/73.1
Hitesiti	Japanese	Small	66.7/80.8	tiestpir sesici	Burmese	Small	48.2/65.4
Maluaba	Japanese	Large	66.7/88.5	resityt setiti	Indonesian	Small	44.4/65.4
Mambuce	Indonesian	Large	66.7/92.3	teiririse siyti	Japanese	Small	44.4/84.6
mhavlema’gam lae	Burmese	Large	66.7/88.5	hisiseiri yteiri	Japanese	Small	37.0/76.9

The size column specifies whether the word is hypothetically congruent with the precision (small) or power grip (large) utilisation. The words in the table are presented orthographically as they were visually presented to the participants. It should be noted that Finnish orthography is very transparent with respect to traditional phonetic transcription with the main differences being letter <ng> for [ŋ], <sh> for [ʃ], and <z> for [ts]. In addition, <c> can be pronounced as [s] or [k]. Consequently, in relation to “small” words, it was pronounced as [s] (i.e., as a precision grip-related consonant), while in relation to “large” words, it was pronounced as [k] (i.e., as a power grip-related consonant). Regarding vowels, the letters <a>, <e>, <i>, <o>, <u>, <y>, and <ä> are pronounced as [ɑ], [e], [i], [o], [u], [y], and [æ], respectively. Thus, for instance, the word *kengagu* was read as [keŋ:ɑgu].

**Figure 1.**
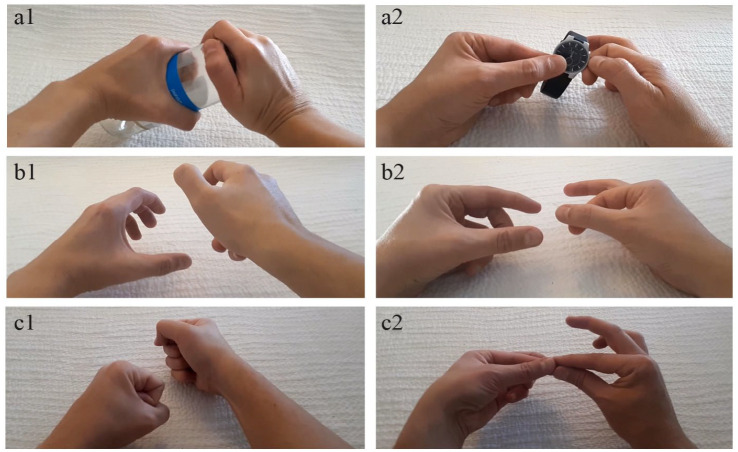
Illustration of stimuli used in Experiment 1 and 2. The image of a1 presents an example of an object-directed WHGU action, a2 presents an example of an object-directed PM action, b1 presents an example of a pantomimic WHGU action, b2 presents an example of a pantomimic PM action, c1 presents an example of a pantomimic WHGU pseudo-action, and c2 presents an example of a pantomimic PM pseudo-action. Experiment 1 employed the stimuli presented in the images of a and b, while Experiment 2 employed the stimuli presented in the images of b and c.

*Visual stimuli*: We also created 48 utilisation videos in which bimanual actions were presented silently from an egocentric perspective (Figure 1). The action movements of each stimulus were executed at approximately the same speed. Twenty-four videos presented PM actions and 24 videos presented WHGU actions (see Table 2 for the actions). The Finnish names of the employed PM actions contained 44 “small” speech sounds and 90 “large” speech sounds, while the Finnish names of the employed WHGU actions contained 56 “small” speech sounds and 65 “large” speech sounds. In two of the precision as well as power videos, the manipulative action was equally executed by the participant’s left and right hands. In the rest of the videos, the right hand was performing a more active role in the manipulation. Half of the precision and power actions were object-directed and half were pantomimes that mimicked the object-directed actions. Pantomimes were created, so that their movement profiles (e.g., kinematic and velocity-related features) were similar in relation to their object-directed counterparts. As previous research demonstrates that jerky and smooth motions as well as wide and narrow motions are sound-symbolically associated with different vowels and consonants (Koppensteiner et al., 2016; Shinohara et al., 2016), the actions were created, so that the profiles of velocity and movement width were similar between the precision and power actions. The mean movement width—measured between the outermost finger locations of the global movement—was 2.7 cm for the precision actions and 2.7 cm for the power actions. The estimation of velocity profiles was based on calculating the action components of each action. For instance, the action of “lighting a match” consisted of two components: taking out the match from the matchbox and lighting the match. Some of the actions consisted of several repetitive hand movements. For example, the action of “cutting a fingernail” consisted of six repetitive movement components. As such, some actions (e.g., “opening a jar” or “lighting a match”) consisted of two slow and smooth hand movements performed at a relatively constant speed, whereas other actions (e.g., “pumping” or “cutting a fingernail”) consisted of several (max. eight) abrupt hand movements. As the length of each action video was identical (4 s), the estimated velocity profile was based on the number of these action components. That is, the more the action video included action components, the more jerky was the velocity profile of the action. The mean of movement components was 3.8 for the precision actions and 4.2 for the power actions.

**Table 2.** The utilisation actions are organised by the size (%) of the congruency effect.

Action	%	Action	%
WHGU/squeezing a dishcloth (O)	87.0	PM/putting on a ring (O)	60.7
WHGU/opening a jar (P)	84.0	PM/threading a needle (O)	60.0
PM/lighting a match (P)	83.3	PM/zipping (P)	60.0
WHGU/squeezing a dishcloth (P)	83.3	WHGU/slicing a cheese (O)	60.0
WHGU/washing a jug (O)	81.3	PM/drawing with a paintbrush (P)	59.4
WHGU/crushing a garlic (P)	80.6	PM/threading a needle (P)	57.9
WHGU/rolling a dough (P)	80.5	PM/tying a thread (P)	56.5
WHGU/brushing a carpet (P)	80.4	PM/stitching (P)	56.5
PM/zipping (O)	78.8	PM/stirring with a teaspoon (O)	55.9
WHGU/slicing a cheese (P)	78.3	PM/opening a small lock (P)	54.6
WHGU/hammering (P)	77.8	WHGU/brushing a carpet (O)	54.1
WHGU/slicing a bread (O)	73.8	PM/stirring with a teaspoon (P)	53.9
PM/tying a thread (O)	73.2	PM/putting on a ring (P)	52.8
WHGU/spraying (P)	68.6	PM/winding a watch (P)	50.0
PM/stitching (O)	68.0	WHGU/grating an apple (O)	50.0
WHGU/opening a jar (O)	68.0	WHGU/hammering (O)	50.0
PM/cutting a fingernail (O)	67.7	WHGU/pumping (P)	50.0
WHGU/grating an apple (P)	66.7	PM/cutting a fingernail (P)	47.7
PM/filing a fingernail (P)	65.9	PM/lighting a matchstick (O)	47.6
WHGU/crushing a garlic (O)	65.6	WHGU/slicing a bread (P)	43.5
WHGU/pumping (O)	64.7	WHGU/rolling a dough (O)	42.1
PM/filing a fingernail (O)	64.3	WHGU/spraying (O)	41.5
WHGU/washing a jug (P)	64.3	PM/winding a watch (O)	33.3
PM/drawing with a paintbrush (O)	61.3	PM/opening a small lock (O)	30.8

PM refers to precision manipulation and WHGU refers to whole-hand grasp usage. The letter O signals that the action was object-directed and the letter P signals that the action was a pantomime.

The experiment consisted of 48 trials. These trials were divided into eight blocks. Each block consisted of three “small” words and three “large” words—presented in a randomised order—selected randomly from a single language category (e.g., Japanese). Each block began by presenting the language category in a written form (e.g., Japanese verbs). Each trial included one PM action video and one WHGU action video. The presentation side of each video was pseudorandomised such that, overall, the same number of videos that sound-symbolically matched with the word were presented on the left and right sides. All of the video pairs in a single language block were presented either as object-directed actions or pantomimes. The words from the same language were presented in two separate blocks: half of them were randomly selected to be presented in relation to object-directed actions and the rest were presented in relation to pantomimic actions. The order of languages was pseudorandomised, so that the same language never appeared in adjacent blocks. Every second block presented object-directed videos, and half of the participants started the experiment with the object-directed videos. Hence, each of 48 words was displayed only once during the experiment, and each of the 48 videos was presented twice during the experiment. However, when the same video was presented the second time, it was never presented in the context of the same language as the first time. The object-directed and the corresponding pantomime action never appeared in adjacent blocks or in the context of the same language.

#### Procedure and apparatus

Each participant sat in a dimly lit room with his or her head 80 cm in front of a 25-inch Full HD monitor (screen refresh rate: 240 Hz; screen resolution: 1920 × 1080). Participants trained for the task in four practice trials. The practice trials presented different words and action videos from those used in the actual experiment. Each verb was auditorily presented over headphones (at ca. 75 dB sound pressure level) three times on the loop with a 1-s gap between each presentation. The written word and the videos were displayed on the loop until the participant pressed the “continue” button. The auditorily presented verb, the written word, and the two action videos were presented at the same time when the participant pressed the continue button. The response was performed by inserting the letter x into the response box that was located under the video (horizontally 16.7°, vertically 9.1°), which was assumed to provide a better match to the word.

The participants were briefed that they were to be presented with foreign verbs in both auditory and written forms in the following languages: Japanese, Burmese, Indonesian, and Hawaiian. They were also told that the verbs were pronounced by a native Finnish speaker (see Table 1 for the used verbs and how they were pronounced in the auditory stimuli) and the pronunciation would therefore not be identical to a native speaker’s pronunciation of the verb. The participants were misinformed that the verb referred to one of the two presented actions in the specified foreign language. They were asked to guess which one of the two videos presented the action to which the verb refers. They were further informed that they could take as much time as they needed to respond. However, they were also advised that there was no reason for them to delay their response if they had a “gut feeling” about the matching action. In that case, they could respond immediately, even before the presentation of the auditory stimulus was finished. The participants were not informed that the pantomimes mimic the presented object-directed actions. However, they were told that the pantomimes mimicked familiar hand manipulation actions.

### Results

To conduct the one-sample *t* test, participants’ responses were converted into percentages of sound-symbolically matching responses separately in relation to the object-directed and pantomime conditions. This test revealed that the participants selected the matching action above the chance level in relation to the object-directed actions, 60.2% (*SD* = 13.3), *t*(26) = 3.98, *p* < .001, *d* = 0.8, as well as pantomimes, 64.5% (*SD* = 11.2), *t*(26) = 6.71, *p* < .001, *d* = 1.3.

The generalised linear mixed model analysis (GLMM) with a binary logistic link function was used to analyse whether these congruency effects differ in relation to the words that are congruent with PM and WHGU actions. The analysis was carried out using the SPSS statistics software package (version 28). In congruent responses (1), the “small” words were matched to PM actions and the “large” words were matched to WHGU actions, while in incongruent responses (2), the “large” words were matched to PM actions and “small” words were matched to WHGU actions. The GLMM analysis treated action category (object-directed vs pantomimes) and word size (“small” words vs “large” words) as fixed factors and subject as well as word as a random intercept. The data are available at https://osf.io/u72qg/.

**Figure 2.**
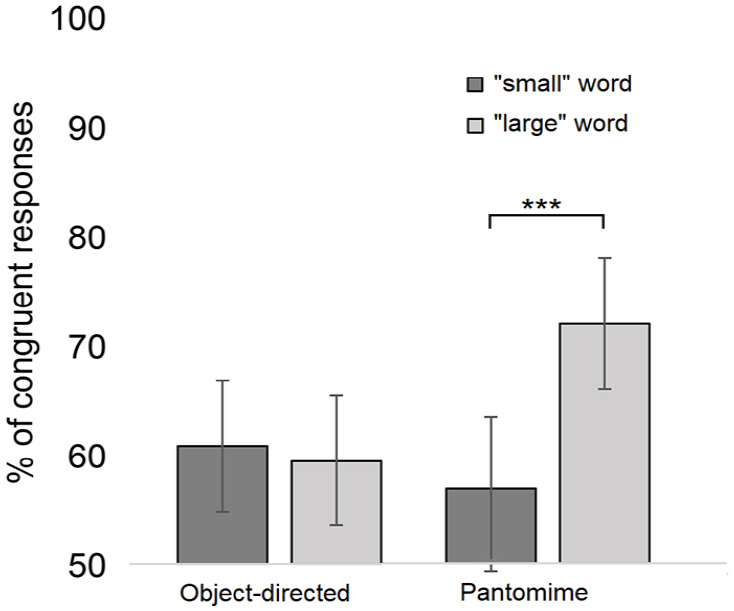
Experiment 1: The mean percentage of sound-symbolically congruent responses as a function of the action category (object-directed vs pantomimes) and word size (“small” words vs “large” words). The asterisks indicate the statistically significant differences (***p < .001). The whiskers represent the confidence intervals (95%) for each condition.

The analysis revealed that word size, *F*(1, 1292) = 4.87, *p* = .028, and word size × action category interaction, *F*(1, 1292) = 10.08, *p* = .002, were significant predictors of the response congruency.^1^ The main effect of Action category was not significant, *F*(1, 1292) = 2.64, *p* = .104. The pairwise comparisons (Bonferroni-corrected) revealed that regarding the pantomime actions, the “large” words were associated with a higher number of congruent responses (*M* = 72.6%; 95% confidence interval [CI] = [66.4%, 78.1%]) than the “small” words (*M* = 56.7%; 95% CI = [49.8%, 63.4%]) (*p* < .001, *d* = 0.99). In addition, the “large” words were associated with a higher number of congruent responses in the pantomime condition (*M* = 72.6%; 95% CI = [66.4%, 78.1%]) than in the object-directed condition (*M* = 59.2%; 95% CI = [52.3%, 65.7%]) (*p* = .001, *d* = 0.82). The corresponding effects for the “small” words (object-directed vs pantomimes: *p* = .263) and object-directed actions (“small” vs “large” words: *p* = .608) were not significant. As also seen in Figure 2, the word size does not influence responses in the object-directed condition, while in the pantomime condition, matching responses are selected more frequently in relation to “large” words than in relation to “small” words.

### Discussion

The results of Experiment 1 showed that the pseudo-words containing “small” speech sounds are associated with the hand actions in which the utilisation is performed using PM action. In contrast, the pseudo-words containing “large” speech sounds are associated with the hand actions in which the utilisation is performed using WHGU action. The participants were most likely to associate the pseudo-words with utilisation actions according to sound-symbolic connections when the word was “large,” and the action presented a pantomime. That is, the sound-symbolic congruency effect appears to be larger with pantomimes than with object-directed actions. Given that pantomimes are recognised by decoding action movements and postures as discussed in the “Introduction” section, it is possible that the sound–action symbolism is mostly based on processing these action properties. However, given that the pantomimes of Experiment 1 copied the object-directed actions of the same experiment, it is possible that this assisted in recognising and processing the pantomimic utilisation actions. That is, we do not know whether the effect could be observed when the actions are not familiar and recognisable. It is, for instance, possible that the effect is boosted by directing the processing resources to the action movements and postures but still requires that the action is recognised in the first place, so that the effect can be observed. Hence, Experiment 2 explores whether the same sound symbolism effect can be observed in relation to the same pantomimic utilisation actions when this benefit is removed by not presenting the object-directed actions among the stimuli set. In addition, Experiment 2 investigates whether this sound symbolism effect can be observed even when the pantomime stimuli do not present any familiar actions. That is, half of the stimuli presented pantomimes of pseudo-actions. In this way, we can investigate whether observing the effect requires that the concrete action is recognised or whether the effect can be observed even when the concrete action is not recognised.

## Experiment 2

### Methods

#### Participants

Twenty-six volunteers participated in Experiment 2 (18–45 years of age; mean age = 27 years; three males and three left-handed). All participants had normal or corrected-to-normal vision and hearing. All participants were native Finnish speakers. In addition, some participants were able to communicate in the following languages: English, Swedish, French, Spanish and Italian. None of them were familiar with any of the following languages: Japanese, Burmese, Indonesian, and Hawaiian. All participants were naive to the purpose of the study; they appeared to be unaware of the purpose of the study and the nature of the investigated effect. We obtained written informed consent from all participants. The study was approved by the Ethical Review Board in Humanities and Social and Behavioural Sciences at the University of Helsinki.

#### Stimuli, procedure, and apparatus

The auditory stimuli of Experiment 2 were the same as that of Experiment 1. In addition, half of the visual stimuli of Experiment 2 consisted of the pantomime action videos used in Experiment 1. The object-directed action videos were replaced by a new set of pantomimic pseudo-action videos in which the action did not imitate any real action. Nevertheless, half of these pseudo-pantomime actions presented PM actions and half of them presented WHGU actions. The mean movement width—measured between the outermost finger locations of the global movement—was 3.7 cm for the PM actions and 4 cm for the WHGU actions. The mean of movement components was 4.7 for the PM actions and 5.5 for the WHGU actions. The apparatus and procedure were the same as in Experiment 1. That is, for example, the real and pseudo-pantomimes were presented in separate sections, so that the pair of action videos contained either real or pseudo-pantomimes. The participants were told that all of the presented videos (i.e., also the pseudo-pantomimes) present a pantomime of a real utilisation action. Finally, after the actual experiment, the participants were presented, one after another, with each action video in randomised order, and they were asked to state if they recognised the action (action recognition test). It was emphasised that if the action did not seem familiar, there was no need to guess the real-life action the pantomime was mimicking. The experimenter wrote down the answers of the participants.

### Results

First, the analysis of the data of the action recognition test showed that, as expected, none of the pseudo-pantomimes were recognised as familiar actions. In contrast, the participants recognised, on average, 45.8% of the real PM pantomimes and 54.2% of the real WHGU pantomimes. It is noteworthy that the action was registered as a recognised action when the guess was close enough to the actual action. That is, for example, “tying a shoelace” and “tying a bow” were both registered as recognised actions in the condition of “tying a knot.”

To conduct the one-sample *t* test, participants’ responses were converted into percentages of sound-symbolically matching responses separately in relation to the real and pseudo-pantomime conditions. This test revealed that the participants selected the matching action above chance level in relation to the real pantomime actions, 73.7% (*SD* = 12.3), *t*(25) = 30.38, *p* < .001, *d* = 1.9, as well as pseudo-pantomimes, 75.8% (*SD* = 12.4), *t*(25) = 30.92, *p* < .001, *d* = 1.9.

The GLMM with a binary logistic link function was used to analyse whether these congruency effects differ in relation to the words that are congruent with PM and WHGU actions. The analysis was carried out using the SPSS statistics software package (version 28). In congruent responses (1), the “small” words were matched to PM actions and the “large” words were matched to WHGU actions, while in incongruent responses (2), the “large” words were matched to PM actions and “small” words were matched to WHGU actions. The GLMM analysis treated action category (object-directed vs pantomimes) and word size (“small” words vs “large” words) as fixed factors and subject as well as word as a random intercept.

**Figure 3.**
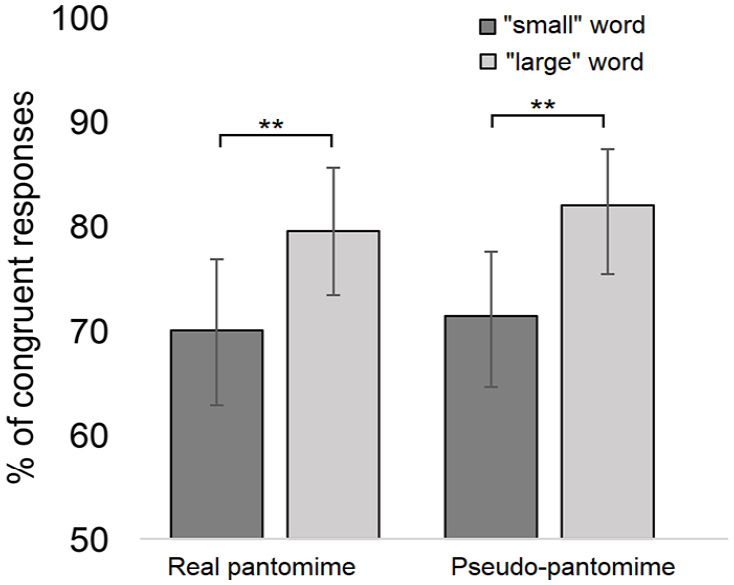
Experiment 2: The mean percentage of sound-symbolically congruent responses as a function of the action category (real vs pseudo-pantomimes) and word size (“small” words vs “large” words). The asterisks indicate the statistically significant differences (**p < .01). The whiskers represent the confidence intervals (95%) for each condition.

The analysis revealed that word size, *F*(1, 1244) = 9.90, *p* = .002, *d* = 0.73,^2^ was a significant predictor of the response congruency. The “large” words were associated with a larger number of congruent responses (*M* = 80.9%; 95% CI = [75.3%, 85.4%]) than the “small” words (*M* = 70.9%; 95% CI = [64%, 76.9%]). The main effect of action category, *F*(1, 1244) = 0.32, *p* = .569, and the interaction between word size and action category, *F*(1, 1244) = 0.01, *p* = .908, were not significant. The pairwise comparisons (Bonferroni-corrected) revealed that this effect was observed in both conditions of visual stimuli (real pantomime: *p* = .018, pseudo-pantomime: *p* = .014). As also seen in Figure 3, the responses are not significantly influenced by the type of visual stimulus (i.e., real vs pseudo-pantomimes).

We also analysed the data of Experiments 1 and 2 in the same analysis to explore whether the sound symbolism effect is different between the four different conditions of action category (i.e., (1) object-directed, 2) real pantomimes of Experiment 1, (3) real pantomimes of Experiment 2, and (4) pseudo-pantomimes). This analysis showed a significant main effect of action category, *F*(3, 2536) = 7.80, *p* < .001. The pairwise comparisons test (Bonferroni-corrected) revealed that the number of congruent responses is significantly smaller in Conditions 1 and 2 in comparison to Conditions 3 and 4 (1 (*M* = 59.7%) versus 3 (*M* = 74.9%): *p* < .001, *d* = 1.14; 1 versus 4 (*M* = 76.8%): *p* < .001, *d* = 1.31; 2 (*M* = 65.9%) versus 3: *p* = .033, *d* = 0.76; 2 versus 4: *p* = .010, *d* = 0.94). That is, the number of congruent responses was significantly larger in the stimulus conditions in which the recognisability of action was relatively weak (i.e., in Conditions 3 and 4).

### Discussion

The results of Experiment 2 showed that the sound-symbolic association between pseudo-words and utilisation actions can be observed in relation to pantomimic hand actions even when the recognition of pantomimes is diminished (i.e., real pantomimes presented without support for recognition from the corresponding object-directed actions) or entirely removed (pseudo-pantomimes). In fact, the effect seems to be even increased in Experiment 2 in comparison to Experiment 1 when the action recognition is diminished or removed. This suggests that sound–action symbolism is not associated with action representations of concrete actions (e.g., lighting a match), but rather it is associated with sensorimotor processes that represent action concepts and/or percepts in a relatively abstract and categorical manner in terms of manipulation types (PM vs WHGU).

## General discussion

It has been suggested that actions might provide a fruitful conceptual context for sound symbolism phenomena (Vainio & Vainio, 2021), even though empirical evidence supporting this view is rather limited. As one of the rare examples of this sound–action symbolism phenomenon, Imai et al. (2008) have previously shown that particular speech sounds are associated with specific manners of walking. To further test this hypothesis, the present study used the 2-AFC task to explore whether the sound-symbolic content of novel words biases the matching of a word to the perceived utilisation action that hypothetically provides the sound-symbolic match to that word. The manipulative hand movements were assumed to present suitable actions for observing the sound–action symbolism effect because a tight coupling between hand and mouth movements has been previously demonstrated (Gentilucci & Corballis, 2006; Rizzolatti et al., 1988; Vainio, 2019). It was assumed that novel words would be intuitively associated with the grasp-related utilisation actions that sound-symbolically match the speech sound content of the word. In line with the hypothesis, the words that were built from “small” speech sounds (e.g., [i], [y], [s], and [t]) were associated with PM actions, while the words that were built from “large” speech sounds (e.g., [a], [o], [k], and [m]) were associated with WHGU actions. This finding presents a novel sound–action phenomenon between speech sounds and tool-use. We propose that the finding supports the proposal (Ramachandran & Hubbard, 2001) that the hand–mouth interaction might manifest itself in sound–action symbolism phenomena in which certain speech sounds are implicitly associated with specific types of grasp- and utilisation-related behaviour.

We have previously proposed (Vainio & Vainio, 2021) that the effect showing coupling between the precision/power grasping and specific vowels and consonants might be based on the overlapping cognitive mechanisms of those presenting sound symbolic association between speech sounds and magnitude dimensions (Sapir, 1929). This can be expected, for example, because the same vowels/consonants that are associated with small/large magnitudes are also associated with precision/power grip responses (Vainio & Vainio, 2022). This assumes that the same processes that carry out abstraction for representing magnitude-related concepts such as small/large, light/heavy, and weak/strong, and associate these concepts with specific speech sounds (Brown et al., 1955; Klank et al., 1971; Osgood et al., 1957), might be responsible for the sound symbolism effect observed in the present study. Furthermore, it has been shown that energetic and heavy manners of walking are sound-symbolically associated with different speech sounds than non-energetic and light manners of walking (Imai et al., 2008; Saji et al., 2013). In the light of these views and evidence, it is possible that when a participant views PM action next to WHGU action, these actions are abstracted to opposite dimensions of weak/forceful, respectively, and these magnitude dimensions are, in turn, sound-symbolically associated with certain speech sounds. However, our view (Vainio & Vainio, 2021) holds that action schemas of grasping and manipulation provide a concrete embodied basis for representing relatively abstract concepts of magnitude, and as such various sound–magnitude associations, are likely to be ultimately based on grounding these abstract concepts of magnitude in concrete action representations of grasping and manipulation.

Interestingly, pantomimes were found to result in even greater sound–action symbolism effects than object-directed utilisation actions. This was particularly the case in Experiment 2—in comparison to Experiment 1—in which the participants were presented with pantomimic action stimuli whose recognisability was either diminished (i.e., real pantomimes presented without support for recognition from the corresponding object-directed actions) or entirely removed (pseudo-pantomimes). This shows that the effect observed in the study is not based on the size of the manipulated object but rather the effect is based on the action that is used to manipulate the object. That is, the sound symbolism effect observed in the present study differs from the previously observed sound–magnitude effects in which actual object size is associated with particular speech sounds (Sapir, 1929; Winter & Perlman, 2021). Furthermore, given that the present study controlled the profiles of velocity and jerkiness between the PM and WHGU stimuli, it seems that the effect is not associated with these movement properties, either. Furthermore, it appears that the sound–action symbolism effect observed in the present study was not based on the processing of a particular concrete action (e.g., opening a jar). Rather, it was based on sensorimotor and/or conceptual processing of viewed actions in a relatively abstract and categorical manner in terms of manipulation types (PM vs WHGU).

One possible reason why the effect was highlighted in relation to the pantomimes is that the effect might originate from the same sensorimotor processes that also enable interpreting and understanding the meaning of iconic gestural signs. Indeed, it has been proposed that pantomimes are essentially communicative in their nature (Arbib, 2005). In line with these views, it has been proposed that the same mechanisms that are responsible for processing iconic gestural signs are also responsible for sound–meaning mappings of spoken language (Dingemanse, 2013; Perniss & Vigliocco, 2014). Both processes are based on representing the meaning of an iconic sign. In line with this, it has been shown that mimetics (i.e., a sound-symbolic word class) are relatively frequently synchronically accompanied by manual gestures (Kita, 1997) in particular when speakers describe motion and action (Kita, 2001). Regarding the present findings, when the task required recognising tool-use actions (Experiment 1), perhaps the cognitive processing was biased towards the context- and object-related recognition processes instead of processing gestural elements of the stimuli, which in turn might have weakened the sound–action symbolism effect. In contrast, when the task required recognising pantomimic utilisation actions whose recognisability was diminished or removed (Experiment 2), the processing was biased to recruit the same sensorimotor processes that are also involved in processing communicative gestures, which in turn might have strengthened the sound–action symbolism effect. Hence, we propose that people might be facilitated to process the meaning of perceived actions and body gestures in the context of language in a sound-symbolic manner.

According to embodied views of cognition, language concepts as well as action semantic knowledge on the use of objects are grounded in motor and perceptual systems (Barsalou, 2008; Pulvermüller, 1999). These accounts can be taken to assume that sound symbolism phenomena—in particular those phenomena that present sound–action correspondences—are similarly grounded in perceptuo-motor processes (Vainio & Vainio, 2021). These embodied views assume that utilisation actions are recognised and understood by the mutually interactive perceptuo-motor processing of action movements as well as the functional and manipulative affordances of an object that is manipulated (Thill et al., 2013). Given this and that interaction occurs between grasping and articulation (Gentilucci & Campione, 2011; Vainio et al., 2013), we propose that the sound-symbolic congruency effect observed in the present study is based on perceptuo-motor processes that integrate perceived utilisation behaviour to corresponding grasp and manipulation-related motor schemas, which in turn are connected to representations of certain articulatory gestures. For example, perceiving a PM action leads to automatically representing this action in the corresponding manual motor processes (e.g., precision grasp) as well as articulatory processes that are connected to these manual processes (e.g., a front-close vowel). This might bias the matching of a presented novel word to the action that is sound-symbolically congruent with the word in the 2-AFC task. For example, when a participant has to decide whether the novel word, which hypothetically contains speech sounds that are linked to the precision grip (e.g., *isytei*), is associated with the presented PM action or the WHGU action, s/he is implicitly primed to select the PM action because articulatory processes that are involved in representing this action are matching with the articulatory processes related to this word.

However, it is important to notify that an equally plausible explanation for the current finding would emphasise acoustic features of the auditory stimuli used in the present study, and sound-symbolic association between these acoustic features and action elements of the visual stimuli. Indeed, for instance, the mainstream explanation of the sound–magnitude symbolism effect (Sapir, 1929) assumes that the effect is based on implicit sound-symbolic mapping of higher/lower frequency speech sounds (e.g., [i] vs [ɑ]) to small/large magnitudes, respectively (Knoeferle et al., 2017; Ohala, 1984). Although we prefer the articulatory account of the effect, this acoustic explanation of the effect cannot be ruled out by the data of the current study. In addition, it is possible that both processes—acoustic and articulatory—contribute to the effect.

The present study had some limitations. First, potential concerns have been raised regarding a forced-choice method. In particular, this method artificially highlights the sound–meaning matches by pre-selecting a few stimuli that are assumed to illustrate the effect of interest (e.g., Westbury, 2005). Therefore, it would be important to replicate the current findings using, for example, an elicitation task in which participants are expected to invent a name for each stimulus using pre-defined consonants and vowels (e.g., Shinohara et al., 2020). Second, although we made an effort to carefully control the motion-related properties of each utilisation stimulus, so that aspects such as the profiles of velocity, jerkiness, and movement width were similar between the precision and power grip-related utilisations, we cannot entirely rule out that some other unknown motion-related feature of the utilisation stimuli might have influenced associating the PM and WHGU actions with different speech sounds. Third, it cannot be inferred from the current results whether the sound-symbolic congruency effect that was observed in the study was based on associating speech sounds with the precision and power grips, with specific types of manipulation actions that only employed these grip types (i.e., PM and WHGU), or with both of these action elements. Finally, the results of the study do not reveal why WHGU pantomimes provided a greater sound symbolism effect than that observed with PM pantomimes. It should be emphasised that the selection of speech sounds for the “small” and “large” words was based on previous knowledge of linkages between particular speech sounds and precision, and power grip responses. However, these grip types can be associated with somewhat different speech sounds than the actions that were included in the present study (i.e., PM and WHGU). Consequently, perhaps the speech sounds that were selected for the “large” words provided a strong match to the WHGU actions, while the speech sounds that were selected for the “small” words provided a somewhat weaker match to the PM actions. This speculation, however, warrants further investigation.

In conclusion, the current study demonstrated that specific speech sounds are sound-symbolically associated with PM and WHGU. This finding supports the view that actions—manual actions in particular—provide a suitable conceptual context for sound symbolism phenomena (Nagumo et al., 2006; Ramachandran & Hubbard, 2001; Vainio & Vainio, 2021). It was also proposed that the same processes that perform abstraction for representing magnitude-related concepts such as light/heavy and weak/strong and link these concepts with specific speech sounds might be responsible for the sound symbolism effect observed in the present study. Moreover, it was proposed that the fact that pantomimes were linked to a greater sound symbolism effect than object-directed actions might suggest that the sound–action symbolism phenomena can be related to the same cognitive processes that enable understanding communicative cues from iconic body gestures.

## Notes

1 When we carried out the analysis including random slopes, the analyses produced the same outcomes as the analyses in which the random slopes were not included.

2 When we carried out the analysis including random slopes, the analyses produced the same outcomes as the analyses in which the random slopes were not included.
